# Investigating associated factors with glomerular filtration rate: structural equation modeling

**DOI:** 10.1186/s12882-020-1686-2

**Published:** 2020-01-29

**Authors:** Parastoo Jamshidi, Farid Najafi, Shayan Mostafaei, Ebrahem Shakiba, Yahya Pasdar, Behrooz Hamzeh, Mehdi Moradinazar

**Affiliations:** 1 School of Medicine, Kermanshah University of Medical Sciences, Kermanshah, Iran; 2 Research Center for Environmental Determinants of Health (RCEDH), Health Institute, School of Health, Kermanshah University of Medical Sciences, Kermanshah, Iran; 3 Epidemiology and Biostatistics Unit, Rheumatology Research Center, Tehran University of Medical Sciences, Tehran, Iran; 4 Nutritional Sciences Department, School of Public Health, Kermanshah University of Medical Sciences, Kermanshah, Iran; 5 Promotion Research Center for Environmental Determinants of Health, Health Institute, Kermanshah University of Medical Sciences, Kermanshah, Iran

**Keywords:** Structural equation modelling, Glomerular filtration, Cohort study

## Abstract

**Background:**

Glomerular filtration rate (GFR) is a valid indicator of kidney function. Different factors can affect GFR. The purpose of this study is to assess the direct and indirect effects of GFR-related factors using structural equation modeling.

**Patients and methods:**

We analyzed data from the baseline phase of the Ravansar Non-Communicable Disease cohort study. Data on socio-behavioral, nutritional, cardiovascular, and metabolic risk factors were analyzed using a conceptual model in order to test direct and indirect effects of factors related to GFR, separately in male and female, using the structural equation modeling.

**Results:**

Of 8927 individuals who participated in this study, 4212 subjects were male (47.20%). The mean and standard deviation of GFR was 76.05 (±14.31) per 1.73 *m*^2^. GFR for 0.2, 11.3, 73.0 and 15.5% of people were < 30, 30 − 59, 60 − 90 and >90, respectively. Hypertension and aging in both sexes and atherogenic factor in males directly, and in females, directly and indirectly, had decreasing effects on GFR. Blood urea nitrogen and smoking in male and female, directly or indirectly through other variables, were associated with a lower GFR. In females, diabetes had a direct and indirect decreasing effect on GFR. Obesity in females was directly associated with upper and indirectly associated with lower GFR.

**Conclusion:**

According to our results, aging, hypertension, diabetes, obesity, high lipid profile, and BUN had a decreasing direct and indirect effect on GFR. Although low GFR might have different reasons, our findings, are in line with other reports and provide more detailed information about important risk factors of low GFR. Public awareness of such factors can improve practice of positive health behaviors.

## Background

Increased prevalence of chronic kidney disease (CKD) is associated with an increased number of deaths as well as other complications in the form of other chronic conditions including cardiovascular diseases. Glomerular filtration rate (GFR) is a valid indicator of kidney function [1, 2]. eGFR has been widely used for the assessment of kidney function as well as to monitor disease progression [3]. In 2013, reduced GFR resulted in 4% (2.2 million) of deaths worldwide, more than half of which caused by cardiovascular (1.2 million people) and endstage renal diseases (ESRD) [4]. The results of clinical trials have shown that decreased GFR is an independent risk factor for all causes of deaths and adverse cardiovascular conditions such as myocardial infarction and stroke [5, 6]. There is also strong evidence suggesting that the development and progression of CKD have been mainly caused by risk factors of cardiovascular diseases including high blood pressure, diabetes, and dyslipidemia. According to the literature, known risk factors for CKD development and progression include aging, diabetes mellitus (DM), hypertension, obesity, dyslipidemia, and smoking [7–13].

Based on the results of previous studies, GFR depends (directly and/or indirectly) on several factors. One of the methods for assessing direct and indirect effects of relevant factors on GFR is the structural equation model (SEM). SEM is one of the most useful methods for the concurrent testing of complex relationships between variables and assessment of the effect of latent variables [14]. SEM is a powerful multivariate analysis method, which allows for the simultaneous verification of a series of regression eqs [15].. This method reduces measurement errors by the involvement of several observed variables for each latent variable. The ability to test the model with several dependent variables and the concurrent direct and indirect effects of several independent variables on the dependent variable are infact amongst the features of SEM. Unlike traditional regression models that treat each covariate in the model as an independent variable with a direct effect on GFR, SEM assesses all pathways of different factors as independent and/or dependent (i.e., mediator) factors. Using SEM, this study aimed to determine the most important risk factors associated with GFR in a group of subjects aged 35_65 who participated in the cohort study of Ravansar. Given the biological and metabolic changes in males and females and the effect of each on the risk factors associated with eGFR, the participants were assessed in both sexes in the present study.

## Material and methods

For the purpose of this study, data from the baseline phase of Ravansar Non-Communicable Disease (RaNCD) cohort study was used. Ravansar, a city in Kermanshah Province is located in the western part of Iran close to the border with Iraq with a population mainly comprised of Kurdish ethnicity. RaNCD cohort is part of the large PERSIAN (Prospective Epidemiological ReSearch in IrAN) study. The data used in this study pertained to more than 10,000 participants aged 35 to 65 who had voluntarily entered the study and signed informed consent forms for participation. The study began in November 2014 and continues to date. Data from the recruitment phase of the study has been collected and includes general data, nutrition questionnaire, and biological samples. More information is available in the cohort protocol [16–18].

### Measurements

Anthropometric indices were determined by bioelectric impedance device. The subjects’ heights were measured by a stadiometer with an accuracy of 1 cm. Body mass index (BMI) was calculated by dividing weight (kg) to squared height (m) [19]. Participants were classified into 5 groups in terms of percent body fat (PBF): 5–10 (Essential Fat), 11–14 (Athletes), 15–20 (Fitness), 21–24 (Average), and 24 > (Obese) for male and 8–15 (Essential Fat), 16–23 (Athletes), 24–30 (Fitness), 31–36 (Average), and 37 > (Obese) for female [20].

Waist to hip ratio (WHR) was classified to either normal or abnormal, according to the third report of the National Cholesterol Education Program (NCEP) on diagnosis, evaluation and treatment of high blood cholesterol in adults (Adult Treatment Panel III) in female and in men [21]. According to guidelines of the international kidney foundation, CKD is defined as renal abnormalities or GFR < 60 ml/min/1.73 (1.0 ml/s/1.73) present for more than 3 months. Renal abnormalities can be diagnosed by pathologic disorders or markers of dysfunction, including abnormalities in blood or urine tests [22]. In this study, Modification of Diet in Renal Disease (MDRD), and nonstandardardized equation (our creatinine values were not standardized for the most part) were used for estimating GFR from age, sex, and creatinine level [23, 24]. Non-use of race variable is due to non-racial differences in this population (almost all participants are from Kurdish ethnicity) eGFR = 1.86 (0.742 if Female) According to CKD Stage cut-point, eGFR was categorized into four groups of > 90, 60–90, 30–59, < 30 ml/min/1.73.To analyze the structural part, eGFR was used as a quantitative variable in the model. Blood pressure was measured after 15 min of rest, twice from the right arm and twice from the left using a sphygmomanometer (RiesterDuplex 1948, Germany). The mean value of the two measurements used as the mean of systolic and diastolic blood pressure. Given the criteria recommended by the Eighth Report of the Joint National Committee on Prevention, Detection, Evaluation and Treatment of High Blood Pressure (JNC-8), people with systolic blood pressure ≥ 140 mmHg and/or diastolic blood pressure ≥ 90 mmHg and/or a history of taking blood pressure-controlling medications were classified as hypertension [25]. Diabetes mellitus (DM) was defined based on the American Diabetes Association’s criteria for fasting blood sugar (FBS) ≤126 Mg /dl and/or patients who used insulin and/or glucose-lowering agents [26]. Smoking was introduced as a self-reported variable (1- none smokers, 2- smokers, 3- former smokers). Blood urea nitrogen (BUN) was calculated based on the quantitative values. Plasma atherogenic index was calculated according to the following formula [27].

$$ \mathrm{Atherogenic}\ \mathrm{coefficient}=\left[\mathrm{Total}\ \mathrm{cholesterol}\right]-\left[\mathrm{HDH}\ \mathrm{cholesterol}\right]/\left[\mathrm{HDL}\ \mathrm{cholesterol}\right] $$

Physical activity calculated according to individual activity per day based on the 22-item questionnaire. Finally, metabolic equivalent of task (MET), as an indicator for level and measure of physical activity, were extracted and entered the model. MET is the amount of oxygen consumed at rest (about 3.5 ml 02/kg/min) and equals to resting metabolic rate. MET for each activity was extracted using compendium of physical activities [28].

Nutritional status was determined according to a valid and reliable food frequency questionnaire customized to the local culture [29]. Consumption of red meat (including red meat, processed meat, liver, heart, gizzard) was another variable derived from food frequency questionnaire calculated based on grams of meat intake per day.

### Statistical methods

Spearman’s rank correlation was applied, and stepwise linear regression was obtained to assess the associations between the study variables and to implement the conceptual framework. Then, structural equation modeling (SEM) was used with maximum likelihood estimation (MLE). SEM includes causal modeling, analysis of covariance structures, and latent variable models. This model is a generalization of multivariate regression that allows one to estimate the strength and sign of direct and indirect effects for complicated causal schemes with multiple dependent and independent variables [30]. In order to create constructs (or factors), we applied confirmatory factor analysis (CFA). CFA is a multivariate statistical technique that is used to test consistency of measures of a construct with the researcher’s understanding of the nature of that construct (or factor). The objective of confirmatory factor analysis is to test whether the data fit a hypothesized measurement model. Path standardized coefficients (β) as the effect sizes of this model were calculated. CMIN/DF) Normed chi-square(,CFI) Comparative fit indices(, GFI)Goodness-of-fit indices(, RMSEA) Root mean squared error of approximation(, NFI) normed fit index (and AGFI)adjusted goodness-of-fitindex (were applied for assessing fitness of the model. Statistical analysis was performed using AMOS-SPSS 22 and STATA 14.0 (STATA Corp, College Station, TX). *P*-value less than 0.05 was considered as statistically significant. As the percentage of missing data was less than 2%, it was excluded from analysis.

## Results

Out of 8927 individuals participated in this study, 4212 (47.20%) subjects were males and 4715 (52.80%) subjects were females. The mean of age was 48.2 ± 2.10 (range:35–65). Prevalence of hypertension in females and males was 16.35 and 10.22%, respectively. Prevalence of BMI was 37% in females and 16.61% in males. The mean of atherogenic coefficient was 187.80 in females and 180.12 in males. Table 1 shows distribution and statistical comparison of the studied variables between four groups of eGFR. The mean ofeGFR was 76.05 ± 14.31 ml/min/1.73. The corresponding values for males and females were80.07 ± 13.89 ml/min/1.73 and 72.46 ± 13.76 ml/min/1.73, respectively. In fact, lower eGFR was associated with older age, hypertension, diabetes, blood lipids, increase in BUN, and lower physical activity (Table 1).

**Table 1:** Comparison of studied variables between four groups of GFR

Variable	Male eGFR (ml/min per 1.73 m2)				*p* value	Female eGFR (ml/min per 1.73 m2)				*p* value
	< 29	30–59	60–90	>90		< 29	30–59	60–90	>90	
Age (mean ± SD)	50.00 ± 10.21	54.00 ± 8.11	47.20 ± 7.90	47.8 0± 7.80	< 0.001	55.90 ± 8.00	52.01 ± 9.01	47.70 ± 8.10	45.70 ± 7.1	< 0.001
BUN (mean ± SD)	37.50 ± 15.48	16.49 ± 4.56	14.9 1± 3.83	14.29 ± 3.82	< 0.001	37.10 ± 24.80	13.61 ± 4.23	12.18 ± 3.51	11.09 ± 3.39	< 0.001
PA (mean ± SD)	35.93 ± 6.99	40.65 ± 8.00	42.70 ± 10.25	42.54 ± 11.19	< 0.001	38.72 ± 4.88	39.77 ± 5.31	39.33 ± 4.51	38.50 ± 3.66	< 0.001
AF (mean ± SD)	171.90 ± 31.57	189.16 ± 39.74	181.24 ± 36.71	173.77 ± 34.84	< 0.001	164.97 ± 31.88	203.58 ± 44.01	185.66 ± 36.75	177.32 ±3 4.84	< 0.001
Red meat (mean ± SD)	21.69 ± 17.14	21.32 ± 28.41	23.64 ± 39.86	21.31 ± 29.29	0.37	18.54 ± 19.30	24.20 ± 35.76	24.61 ± 36.22	22.70 ± 28.98	0.37
Organ meat (mean ± SD)	2.90 ± 1.62	5.02 ± 10.01	4.46 ± 9. 48	4.20 ± 8.10	0.02	1.35 ± 1.37	5.69 ± 15.51	5.02 ± 9.76	4.62 ± 8.57	0.02
Process meat (mean ± SD)	1.23 ± 2.01	2.50 ± 7.71	1. 9 3± 6.01	1.89 ± 7.64	0.005	0.32 ± 1.02	3.04 ± 9.84	2.26 ± 6.83	2.51 ± 7.58	0.005
BMI,n (%)										
≤ 18.4	0(0.0%)	1(0.48%)	66(2.11%)	20(2.29%)	0.5	0(0.0%)	13(1.61%)	50(1.47%)	3(0.58%)	0.006
18.5–24.9	3(37.50%)	76(36.89%)	1080(34.54%)	315(36.12%)		1(10.00%)	215(26.70%)	700(20.66%)	108(21.09%)	
25.0–29.9	4(50.00%)	86(41.74%)	1451(46.41%)	411(47.13%)		4(40.00%)	326(40.49%)	1390(41.02%)	205(40.03%)	
30.0–34.9	1(12.50%)	37(17.96%)	463(14.81%)	106(12.15)		5(50.00%)	187(23.22%)	937(27.65%)	159(31.05)	
≥ 35	0(0.0%)	6(2.91%)	66(2.11%)	20(2.29%)		0(0.0%)	64(7.95%)	311(9.17%)	37(7.22%)	
PBF, n (%)										
5–10	0(0.0%)	1(0.50%)	27(0.90%)	7(0.80%)	0.2	0(0.0%)	1(0.12%)	4(0.11%)	2(0.40%)	0.009
11–14	0(0.0%)	3(1.45%)	116(3.71%)	37(4.24%)		0(0.0%)	26(3.22%)	74(2.18%)	7(1.36%)	
15–20	0(0.0%)	30(14.56%)	462(14.77%)	122(13.99%)		0(0.0%)	80(9.93%)	241(7.11%)	31(6.05%)	
21–24	0(0.0%)	40(19.41%)	444(14.20%)	106(12.15%)		2(20.00%)	187(23.22%)	681(20.10)	97(18.94%)	
> 24	8(99%)	132(64.07%)	2077(66.44%)	600(68.80%)		8(80%)	511(63.47%)	2388(70.48%)	375(73.24%)	
WHR, n (%)	3(37.50%)	81(39.32%)	1191(38.09%)	335(38.41%)	0.9	6(60.00%)	601(74.65%)	2591(76.47%)	384(75.00%)	0.4
BP, n (%)	4(50.00%)	63(30.58%)	298(9.53%)	65(7.45%)	< 0.001	6(60.00%)	230(28.57%)	495(14.61%)	43(8.39%)	< 0.001
Diabetes,n (%)	0(0.0%)	37(17.96%)	243(7.77%)	58(6.65%)	< 0.001	1(10.00%)	93(11.55%)	272(8.02%)	31(6.05%)	< 0.001
Smoking, n (%)										
No smoker	5(62.50%)	130(63.10%)	1993(63.75%)	557(63.87%)	< 0.001	8(80.00%)	725(90.06%)	3226(95.21%)	500(97.65%)	< 0.001
Current smoker	1(12.50%)	31(15.04%)	701(22.41%)	217(24.88%)		0(0.0%)	32(3.97%)	61(1.80%)	4(0.78%)	
Former smoker	2(25.00%)	45(21.84%)	432(13.81%)	98(11.23%)		2(20.00%)	48(5.96%)	101(2.598%)	8(1.56%)	

**Fig. 1:**
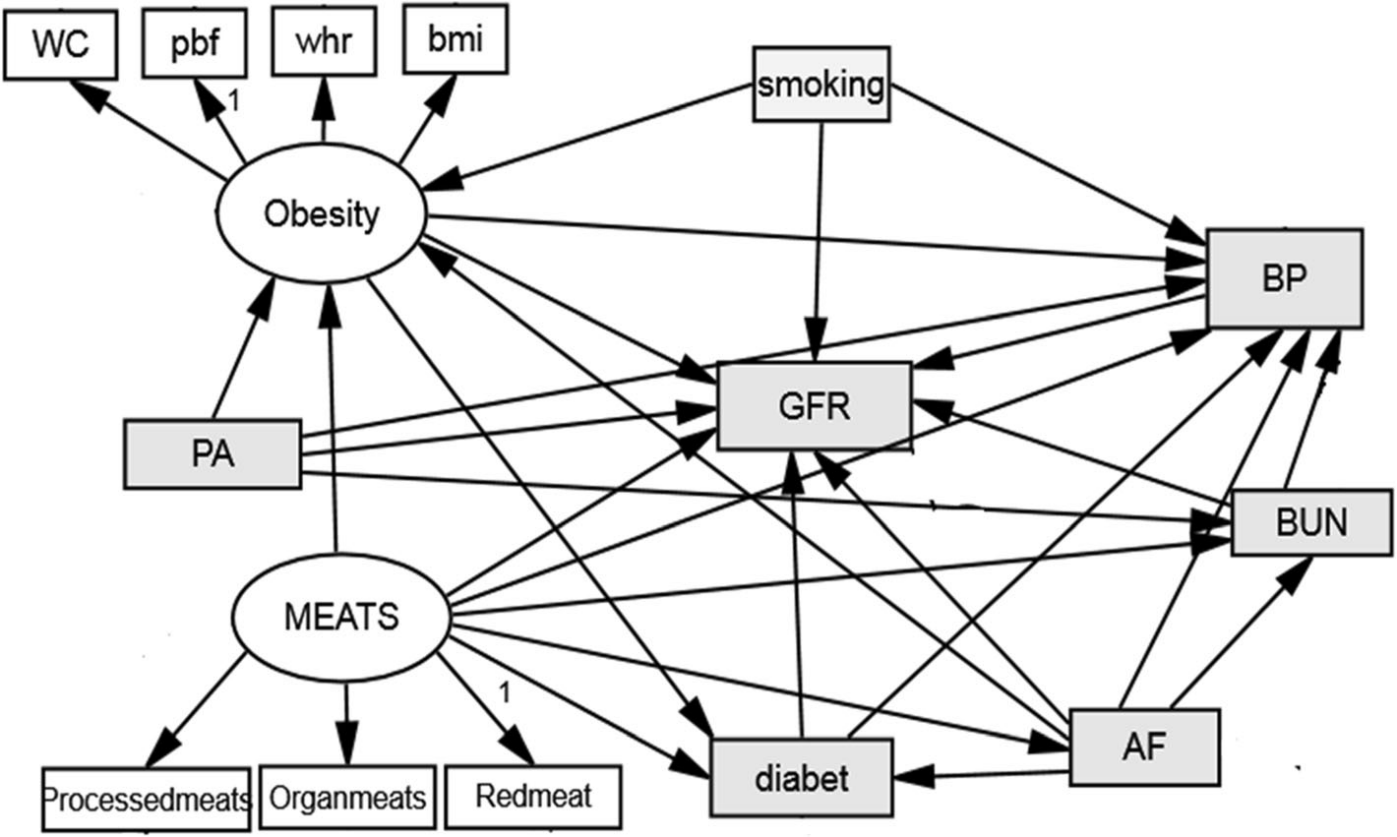
The conceptual model diagram for risk factors relationship with glomerular filtration rate. BUN, blood urea nitrogen; PA, physical activity; BP, blood pressure; AF, atherogenic factor, WHR; waist to hip ratio; BMI, body mass index; PBF, percent body fat

Structural equation modeling (SEM) with maximum likelihood estimation (MLE) was applied to assess the conceptual model (Fig. 1). Confirmatory Factor Analysis (CFA) was used to confirm a group of variables with an internal consistency with a latent variable. Waist circumference was removed from the model due to low loading factor and poor fitting. For other variables, applied CFA goodness of fit indices were at appropriate levels (CMIN/DF: 1.19, GFI: 0.99, RMSEA: 0.005, CFI: 0.99). These indices indicated acceptable fitting of the model.

Table 1 The outside the parentheses is the number of people, and the values inside the parentheses are the percentages. Data are expressed as mean and SD. *P* values were estimated using tow-way analysis variance or test. BUN: Blood Urea nitrogen; PA: physical activity; BP: Blood pressure; AF: atherogenic Factor; WHR: Waist to hip ratio; BMI: Body mass index; PBF: Percent body fat; GFR: glomerular filteration rate.

**Fig. 2:**
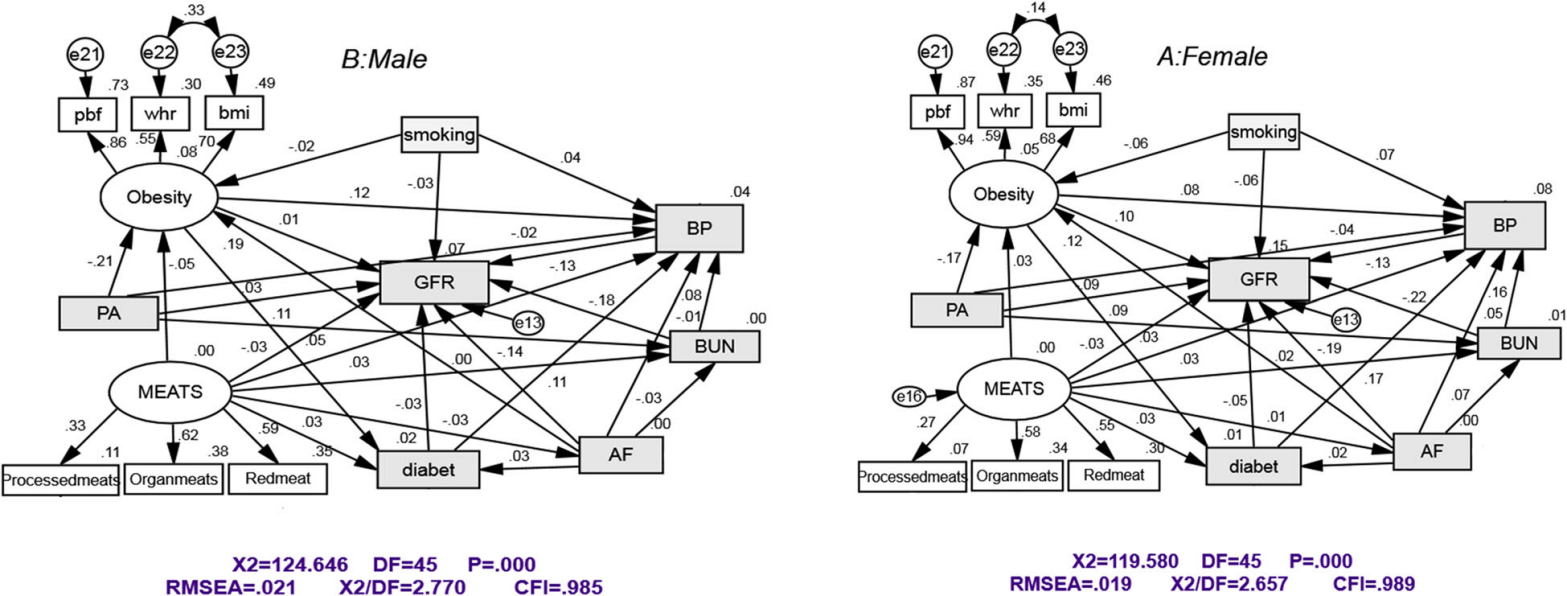
Part **a** and **b**: shows structural equation models for assessing direct and indirect effects on GFR for both females and males by standardized path coefficient and goodness of fit indices.”e” represent the errors. BUN, blood urea nitrogen; PA, physical activity; BP, blood pressure; AF, atherogenic factor WHR, waist to hip ratio; BMI, body mass index; PBF, percent body fat

Figure 2 Part A and B shows structural equation models for assessing direct and indirect effects of GFR for both females and males by standardized path coefficient and goodness of fit indices.”e” represent the errors. Note. BUN: blood urea nitrogen; PA: physical activity; BP: blood pressure; AF: atherogenic factor WHR: waist to hip ratio; BMI: body mass index; PBF: percent body fat

For females, atherogenic index had a direct (β =  − 0.19) and indirect (β =  − 0.01) decreasing effects via BUN, high blood pressure, diabetes and obesity as an intermediate variables on eGFR. BUN had direct (β =  − 0.22) and indirect (β =  − 0.02) decreasing effects via hypertension on eGFR. Hypertension was associated with lower eGFR (β =  − 0.12). Diabetes had direct (β =  − 0.04) and indirect (β =  − 0.02) decreasing effects on eGFR. Obesity had direct positive (β = 0.10) and indirect negative (β =  − 0.02) effects on eGFR. Diabetes and BUN were associated with high blood pressure (β = 0.17 and β = 0.15, respectively). For males, atherogenic index had a direct negative effect (β =  − 0.13) on GFR. BUN had direct (β =  − 0.17) and indirect (β =  − 0.01) negative effects via high blood pressure, as an intermediate variable, on GFR. Hypertension decreased directly (β =  − 0.12) GFR. Smoking had a direct and indirect decreasing effect (β =  − 0.02 and β =  − 0.005) effects, via obesity and high blood pressure, as intermediate variables, on GFR. Physical activity had a direct negative (β =  − 0.20) effect on obesity . Meat consumption in females had a direct (−0.01) and indirect (−0.03) effect on GFR (no effect on eGFR in males) (Table 2). All goodness of fit indices indicated that the model has acceptable fit. The results of the model fitness were reported in Fig. 2.

**Table 2:** SEM results in 35–65-year-old- by sex at RaNCDchort study

Variable	Male			Female		
	Total effect (95%CI)	Direct effect (95%CI)	Indirect effect (95%CI)	Total effect (95%CI)	Direct effect (95%CI)	Indirect effect (95%CI)
GFR > --- Smoking	0.03- (0.06-, 0.005 -)	−0.02 (− 0.05, − 0.01)	− 0.005 (− 0.01, − 0.001)	− 0.07 (− 0.10, − 0.05)	--0.06 (− 0.08, − 0.03)	−0.01 (− 0.02و -0.009)
AF --- > GFR	−0.13 (− 0.16, − 0.10)	− 0.13 (− 0.16, − 0.10)	–	−0.20 (− 0.23, − 0.17)	−0.19 (− 0.23, − 0.17)	− 0.01 (− 0.02, − 0.03)
BUN --- > GFR	−0.18 (− 0.23, − 0.14)	−0.17 (− 0.22, − 0.13)	−0.01 (− 0.01, − 0.006)	−0.24 (− 0.28, − 0.20)	− 0.22 (− 0.26, − 0.18)	0.02- (− 0.02, − 0.01)
HTN--- > GFR	−0.12 (− 0.15, − 0.09)	− 0.12 (− 0.15, − 0.09)	–	− 0.12 (− 0.15, − 0.09)	− 0.12 (− 0.15, − 0.09)	–
Meats--- > GFR	–	–	–	−0.04 (− 0.08, − 0.001)	−0.01 (− 0.02, 0.003)	−0.03 (− 0.06, 0.005)
Obesity--- > HTN	0.13 (0.10, 0.17)	0.12 (0.08, 0.15)	0.01 (0.007, 0.02)	0.09 (0.06, 0.12)	0.07 (0.04, 0.10)	0.02 (0.01, 0.03)
Obesity--- < GFR	–	–	–	0.08 (0.07, 0.13)	0.10 (0.007, 0.13)	−0.02 (− 0.02 و -0.01)
Diabetes--- > GFR	–	–	–	−0.06 (− 0.09, − 0.03)	0.04- (− 0.07, − 0.01)	−0.02 (− 0.02, − 0.01)
Obesity--- > Diabetes	0.11 (0.08, 0.14)	0.11 (0.08, 0.14)	–	0.08 (0.06, 0.11)	0.08 (0.06, 0.11)	–
Diabetes--- > HTN	0.11 (0.06, 0.15)	0.11 (0.06, 0.15)	–	0.17 (0.14, 0.21)	0.17 (0.14, 0.21)	–
P A--- > Obesity	−0.20 (− 0.24, − 0.17)	− 0.20 (− 0.24, − 0.17)	–	−0.16 (− 0.20, − 0.13)	− 0.16 (− 0.20, − 0.13)	–
Lipid profile --- > Obesity	0.19 (0.15, 0.22)	0.19 (0.15, 0.22)	–	0.13 (0.10, 0.16)	0.13 (0.10, 0.16)	–
BUN --- > HTN	0.07 (0.04, 0.11)	0.07 (0.04, 0.11)	–	0.15 (0.12, 0.18)	0.15 (0.12, 0.18)	–

*BUN* blood urea nitrogen, *PA* physical activity, *BP* blood pressure, *AF* atherogenic factor, *WHR* waist to hip ratio, *BMI* body mass index, *PBF* percent body fat. Interpretation of one result as an exemple: In female, atherogenic variable had direct (β =  − 0.19) and indirect (β =  − 0.01) decreasing effects via mediating variables (BUN, high blood pressure, diabetes and obesity) on GFR

## Discussion

In this population-based study, we examined factors associated with glomerular filtration rates (GFR) in both genders. The findings of our study showed that obesity, diabetes, blood urea nitrogen, atherogenic factor, hypertension, meat consumption, and smoking were associated with lower GFR.

Several risk factors (hypertension, diabetes, high blood lipids and smoking) can affect on eGFR which has been reported from studies elsewhere [31]. Multivariate analysis of a retrospective cohort study on patients with renal disease in Japan (2012) showed that smoking, high blood pressure, high triglycerides, and low HDL each had an independent effect on CKD. Other studies also showed similar results regarding the effects of hypertension and high TG and LDL levels on CKD [32, 33].

In the current research, obesity in females had direct (positive) and indirect (negative) effects, via hypertension and diabetes, on GFR. Results from studies on the effect of obesity on GFR are not similar [34, 35]. Iseki et al. reported an independent relationship between obesity and ESRD [11]. Like obese people, overweight people were more likely to develop ESRD [36]. Hypotheses suggest that low muscle mass is associated with low levels of serum creatinine, resulting in low GFR in normal people with no CKD. Nonetheless, obesity increases the risk for type 2 diabetes, hypertension and dyslipidemia [37], which in turn lead to low GFR.

A meta-analysis showed that there is a U-shape relationship between eGFR and death rate; eGFR< 60 ml/min/173 m2 increases death rate incrementally, but eGFR> 105 ml/min/173 m2 results in a sharp decrease in death rate [38].

In the RaNCD cohort study, low value of eGFR in females was due to inadequate physical activity and high prevalence of metabolic risk factors such as obesity, high blood lipids, and hypertension. eGFR was also related to BUN which had a negative direct and indirect relationship with GFR in both males and females. The value for BUN is, in fact, a sign of proper kidney functioning. The main causes of increased BUN are high-protein diets, low GFR, and congestive heart failure. An increase in BUN may be independent of changes in creatinine and GFR. Such increase is due to reabsorption from proximal tube through the activity of renin-angiotensin-aldosterone sympathetic nervous systems [39]. There is a non-linear relationship between increasing BUN and decreasing GFR. Significant GFR decrease (> 75%) is associated with an increase in BUN in the early stage of a renal disease. On the other hand, a relatively minor decrease in GFR is associated with a relatively high increase in urea concentrations and serum creatinine [40]. In our study, BUN had a negative and indirect effect via high blood pressure, as an intermediate variable on GFR. Findings of previous studies suggested that high blood pressure is significantly associated with increased kidney damage in females and males [41, 42]. A meta-analysis study in 2014 showed a significant relationship between high blood pressure and incidence of ESRD [43]. In the current research, hypertension was directly related to decrease in GFR. Meat consumption in women has a direct and indirect effect on GFR, which is consistent with similar studies [44, 45].

Dyslipidemia is an important risk factor for cardiovascular disease and CKD. In a study of 12,728 subjects with a 2-year follow up, it was found that high triglyceride and low HDL both were risk factors for increased creatinine. These lipid profiles had confusing effects on creatinine after adjustment for other risk factors [43]. The mechanism through which fat causes damage to kidneys is not clear, but glomeruli sclerosis and atherosclerosis seems to have similar effects [46]. The current research findings showed that atherogenic index had direct and indirect relationship with low levels of GFR.

It is worth noting that this study is the first study that uses SEM for assessing the risk factors associated with GFR. The most important strength of the present study was the sample size which was large enough to investigate the association between all the above-mentioned variables with GFR. Using SEM and path analysis, we were able to investigate both direct and indirect effects of GFR risk factors. However, our study suffered from the following.

### Limitations

Using a cross-sectional study, we were unable to confirm that the studied exposures had an exact causal relationship with the level of eGFR. The researchers’ definition of eGFR was only based on serum creatinine criterion which could lead to biased classification. Other studies have shown that eGFR measurement for subjects with normal kidney functioning was performed with less accuracy than those with CKD. Nevertheless, it was more accurate than serum creatinine or Cockcroft-Gaultequation.

## Conclusion

Findings of the present study confirmed the results of previous studies on the risk factors of eGFR including hypertension, diabetes, blood lipids, BUN, obesity and smoking. Although low eGFR might have different reasons and is not a consistent sign of CKD, our findings are in line with reports from elsewhere and provides more detailed information about important risk factors of low GFR. Awareness about such risk factors will lead to positive health behavior in general public. Future studies are recommended to investigate the effect of other variables including medications and food on eGFR.

## Data Availability

All the information on how to access the RaNCD, with a list of current proposals and papers currently under preparation, can be found on our website: www.persiancohort.com.

## Abbreviations

AF: Atherogenic factor
AGFI: Adjusted goodness-of-fitindex
BMI: Body mass index
BUN: Fasting blood sugar (FBS), Blood urea nitrogen
CFA: Confirmatory factor analysis
CFI: Comparative fit indices
CKD: Chronic kidney disease
CMIN/DF: Normed chi-square
DM: Diabetes mellitus
ESRD: End-stage renal diseases
GFI: Goodness-of-fit indices
GFR: Glomerular filtration rate
MDRD: Modification of Diet in Renal Disease
MET: Metabolic equivalent of task
MLE: Maximum likelihood estimation
NCEP: National Cholesterol Education Program
NFI: Normed fit index
PBF: Percent body fat
PERSIAN: Prospective Epidemiological ReSearch in IrAN
RaNCD: Ravansar Non-Communicable Disease
RMSEA: Root mean squared error of approximation
SEM: Structural equation model
WHR: Waist to hip ratio
